# Reduced turnover times make flexible optical reusable scope with EndoSheath^®^ Technology significantly cost-effective

**DOI:** 10.7555/JBR.26.20120050

**Published:** 2012-07-08

**Authors:** Deepak Gupta, Arvind Srirajakalidindi, Hong Wang

**Affiliations:** Department of Anesthesiology, Detroit Medical Center, Wayne State University, Detroit, MI 48201, USA

**Keywords:** Olympus scope, EndoSheath^®^ Technology, intubation cost-analysis, turnover times

## Abstract

EndoSheath bronchoscopy (Vision Sciences, Inc.) uses a sterile, disposable microbial barrier that may meet the growing needs for safe, efficient, and cost effective flexible bronchoscopy. The purpose of this open-label comparative study was to compare and calculate the costs-per-airway-procedure of the reusable fiberscope when used with and without EndoSheath^®^ Technology; and to record the turnover time from the completion of the use of each scope until its readiness again for the next use. Seventy-five new patients' airways requiring airway maneuvers and manipulations with Vision Sciences, Inc., reusable fiberscope with EndoSheath^®^ Technology were evaluated for the costs comparisons with reassessed historical costs data for Olympus scope assisted tracheal intubations. As compared to costs of an intubation ($158.50) with Olympus scope at our institute, the intubation costs with Vision Sciences, Inc., reusable fiberscope with EndoSheath technology was $81.50 (*P* < 0.001). The mean turnover time was 5.44 min with EndoSheath technology as compared to previously reported 30 min with Olympus fiberscope (*P* < 0.001). Based on our institutional experience, Vision Sciences, Inc., reusable fiberscope with EndoSheath technology is significantly cost effective as compared to the Olympus scope with significantly improved turnover times.

## INTRODUCTION

Flexible fiberoptic scopes used for endotracheal intubation have been limited due to its high cost of purchase and disinfection, and special skills needed by the operator for its fast and effective use in critical scenarios of airway management. There has been recent documentation of the microbial spectrum that has to be resolved for proper disinfection of the bronchoscopes[1]. Moreover, the sterilization processes, which are more advanced than routine disinfection processes, are desired when performing bronchoscopy in immunocompromised patients because they are critical and act as sporicidal prevention of the transmission of spores to these immunocompromised patients. When the sporicidal sterilization processes are not feasible, even high level disinfection (elimination of most pathological organisms except spores) of these scopes is also tedious and cumbersome, further increasing the cost of intubation and decreasing the turnover time for its rapid use between cases.

In reality, there has always been an urgency to improve the design of fiberscope to overcome the deadly issues of cross contamination and infectious outbreaks[2]-[4] in hospital settings, including transmission of prion disease[5]. To address these issues, there have been two new commercially available additions to aid intubations: Single use scopes[6] and Reusable scopes with EndoSheath^®^ Technology[7]. The cost of the single use scope may be a major deterrent to the rapid replacement of the reusable scopes with single use scopes[8]. Additionally, it remains to be seen if the camera technology used in single use intubation scopes is as flexible as reusable fiberoptic intubation scopes when used in difficult airways and how the absence of the suction channel in the single use intubation scope usually interferes with successful intubation. This absence of suction channel in the single use scope can be overcome with the use of the EndoSheath^®^ Technology with suction channels. EndoSheath^®^ Bronchoscopy (Vision Sciences, Inc.), which uses a sterile, disposable microbial barrier, provides anesthesiologists with a solution to meet the growing need for safe, efficient, and cost effective flexible bronchoscopy. EndoSheath^®^ Technology is a sterile, disposable sheath that covers the bronchoscope and completely isolates it from patient contact during the procedure. Each sheath incorporates a disposable channel for suction, fluid introduction, and tool passage. All patient materials, such as blood, biopsies, and fluids, only come in contact with the disposable sheath and never the reusable bronchoscope. Additionally, environmental waste management is less cumbersome with the disposal of EndoSheath^®^ Technology as compared to the disposal of single use scope. However, there has been no cost study comparing the use of classical reusable scopes and the D-shaped reusable scope covered with the EndoSheath^®^ Technology. The purpose of this study was to compare and calculate the costs per airway procedure of the reusable fiberscope when used with and without EndoSheath^®^ Technology, and to record the turnover time from the completion of the use of each scope until its readiness again for the next use.

## MATERIALS AND METHODS

### Protocol of study

After institutional review board approval, the present study that was co-sponsored by Vision Sciences, Inc., Orangeburg, New York, United States, was conducted at Department of Anesthesiology of a major Academic University Hospital. The study was designed as an open-label observational study to compare cost data[8] for Olympus reusable fiberscope (that does not have EndoSheath^®^ Technology) with new patients' cost data whose tracheas were intubated with the Vision Sciences, Inc., reusable fiberscope with EndoSheath^®^ Technology. After approval of the Hospital Committee, 75 new patients undergoing procedures requiring airway maneuvers and manipulations with fiberoptic scope were included in the study so that they could undergo airway procedure assisted with Vision Sciences, Inc., reusable fiberscope with EndoSheath^®^ Technology to evaluate the airway procedure costs. Subsequently, turnover times for the reusable scope with EndoSheath^®^ Technology were recorded as the time periods from the end of an airway related procedure to the scope's readiness (after cleaning of the used scope and re-loading of the new EndoSheath^®^ on the cleaned scope) for the next airway related procedure. Thereafter, the procedural costs of EndoSheath^®^ Technology were adjusted based on the comparative turnover times (recorded turnover time of EndoSheath^®^ Technology versus previously reported turnover times of Olympus Scope). Finally, the procedural costs and turnover times were statistically compared between the Olympus reusable fiberscope (that does not have EndoSheath^®^ Technology) with the Vision Sciences, Inc., reusable fiberscope with EndoSheath^®^ Technology.

For comparison of the previously reported costs after reassessment[8], the costs to the reusable scope with EndoSheath^®^ Technology were accrued as following: Based on the changed turnover times and manufacturer's recommended life span of the scope (9 years), cost of acquiring reusable scope with EndoSheath^®^ Technology; based on the changed turnover times, number of reusable scope with EndoSheath^®^ Technology required; annual full maintenance contract (the full coverage of the repairs) for the reusable scope with EndoSheath^®^ Technology; total disinfecting materials costs and personnel work hours billed for the OR personnel involved regarding cleaning and preparing the scopes, disinfecting the scope if there was a breach in the used EndoSheath^®^, and transporting the scopes.

Per manufacturers' recommendations, the reusable scope with EndoSheath^®^ Technology was manually washed with EndoWipe^®^ Enzymatic Cleaning Sponge and running water, and then dry cleaned with EndoWipe^®^ Towelette. If there was a breach appreciated in the EndoSheath^®^ (as determined by visual inspection for the presence of moisture on the surface of the insertion cord of the reusable scope after removal of the used EndoSheath^®^), the standard protocol of cleaning and disinfecting the reusable scope[8] was followed.

### Statistical ananlysis

Data were expressed as mean±SD and analyzed using the SPSS software Version 13.0 (SPSS Inc., Chicago, IL,USA). Two-tailed Chi-square test was used for the statistical significance and the values of *P* < 0.05 were considered significant.

## RESULTS

A total of 75 airway related procedures *(****Table 1****)* were performed with Vision Sciences, Inc., reusable fiberscope with EndoSheath^®^ Technology. The attempts at the airway related procedures were limited to one attempt with reusable fiberscope with EndoSheat^®^ Technology; decision to use any other airway instrument for subsequent attempts were left at the discretion of the supervising anesthesia providers and their team members. The overall success rate at the first attempt completion of the intended airway related procedure was 83% with EndoSheath^®^ scope that is comparable to our success rates with Olympus scope. During the cleaning of the scope between the procedures, not a single breach of the EndoSheath^®^ was appreciated and hence after each of the 75 patient-use, cleaning of the reusable fiberscope with EndoSheath^®^ Technology was limited to only manually washing with EndoWipe^®^ Enzymatic Cleaning Sponge and running water, and then dry cleaning with EndoWipe^®^ Towelette. The turnover time for reusable fiberscope with EndoSheath^®^ Technology was 5.44±0.63 min.

As our previously reported data[8] was related to the annual 166 tracheal intubations performed with the available six Olympus fiberoptic reusable scopes, the calculations were initially matched to the 166 intubations with six scopes for calculating the raw data for EndoSheath^®^ Technology. Subsequently, based on the comparative turnover times between the Olympus Scope (30 min) versus the EndoSheath^®^ Technology (5.44 min), the equivalent requirements of the Vision Sciences, Inc., reusable fiberscope with EndoSheath^®^ Technology were calculated as 1.09 Scopes [(6 Scopes/30 min)(5.44 min)]. As the Vision Sciences, Inc., reusable fiberscope with EndoSheath^®^ Technology number should be logically and tangibly a whole number to match up the facilities requirements for anesthesia related airway procedure inside and outside the operation room suites, the final calculation was based on the minimum need of two EndoSheath^®^ Technology scopes at our facility to match the annual 166 intubations with six Olympus scopes. As explained and documented in ***Table 2***, the procedural cost calculations for Vision Sciences, Inc., reusable fiberscope with EndoSheath^®^ Technology were done as follows: Six EndoSheath^®^ Technology Scopes (BRS-4000 Flexible Fiber Bronchoscope) purchase prices are $11,650.00 each (Total amount for purchasing six EndoSheath^®^ Technology Scopes is $69,900.00). Recommended average lifetime of the EndoSheath^®^ Technology Scopes is 9 years. Thus, the annual cost of purchasing six scopes is $7,766.67 [(11,650/9)(6)]. Based on the previously reported data based on the annual 166 intubations performed with Olympus LF-GP Scopes, the equivalent cost of purchasing EndoSheath^®^ Technology Scopes for each intubation is $46.79 (7,766.67/166). As the EndoSheath^®^ Technology Scopes offer an annual full maintenance contract (to cover all the repairs) for the scopes at $4,000.00 per scope, the equivalent repair cost per intubation with six EndoSheath^®^ Technology Scopes are $144.58 [(4,000/166)(6)].

**Table 1:** Airway procedure and the first attempt success rate of Vision Sciences, Inc., reusable fiberscope with EndoSheath^®^ Technology

Fiberoptic guided airway procedures	Attempts			Reasons for failures (n)
	Total	Successful	Failed	
Sleep fiberoptic tracheal intubations	60	49	11	Failed visuals (6) Failed to intubate (4) Patient vomited (1)
Awake fiberoptic tracheal intubations	2	2	-	-
Assisting GlideScope intubations	3	3	-	-
Double lumen endobronchial tube placements	7	5	2	Poor lubrication (1) Larger tube size (1)
Bronchoscopy	2	2	-	-
Broncho-alveolar lavage	1	1	-	-
Total	75	62	13	Overall success rate 83%

**Table 2:** Cost-analysis of the Vision Sciences, Inc., reusable fiberscope with EndoSheath^®^ Technology and its comparison with the previously reported data of Olympus fiberscope

Materials that incurred costs	Annual amounts (in USD, based on six scopes Olympus)	Cost per intubation (in USD, based on historical 166 annual intubations)	Annual amounts (in USD, matched to six scopes EndoSheath^®^)	Cost per procedure (in USD, matched to historical 166 annual Intubations EndoSheath^®^)	Cost per procedure (in USD, matched to mean turnover times elicited equivalent 1.09 scopes EndoSheath^®^)	Cost per procedure (in USD, matched to labor logical tangibility elicited equivalent two scopes EndoSheath^®^)
Purchase	5,766.67	34.74	7,766.67	46.79	8.50	15.60
[(Cost of Scope/Lifetime of scope) (number of scopes)]	[(8650/9)(6)]		[(11650/9)(6)]			
					
Annual Maintenance Contract	12,888.00	77.64	24,000.00	144.58	26.27	48.19
	[(2148)(6)]		[(4000)(6)]			
Disinfection	5505.60	33.16	7,053.34	42.49	42.49	42.49
Labor	2150.63	12.96	703.20	4.24	4.24	4.24
TOTAL	26,310.90	158.50	39,523.21	238.10	81.50	110.52
				*P* = 0.007	*P* < 0.001	*P* = 0.009
				(Favoring Olympus)	(Favoring EndoSheath)	(Favoring EndoSheath)

The costs of cleaning and preparing the EndoSheath^®^ Technology Scopes are $42.49, as following details: Use of different sizes of the single use (disposable sheaths) BF-0/BF-1.5/BF-2.1 EndoSheath^®^ Technology for the BRS-4000 Flexible Fiber Bronchoscope with each airway procedure, Cost: $40.00 per sheath; Washing of the BRS-4000 Flexible Fiber Bronchoscope with one EndoWip^®^ Enzymatic Cleaning Sponge after each airway procedure, EndoWipe^®^ Enzymatic Cleaning Sponge comes in the configuration of 20/box-4 boxes/case and the price of a case is $175.00. Cost: $2.19 per intubation (175/80); Dry Cleaning of the BRS-4000 Flexible Fiber Bronchoscope with one EndoWipe^®^ Towelette after washing with sponge and running water, EndoWipe^®^ Towelettes come in the configuration of 50/Tub-12 tubs/case and the price of a case is $180.00. Cost: $0.30 per intubation (180/600).

The personnel hours are billed at $14.00 per hour with 30% fringe benefit. Based on the previously reported data[8] of the intubation scope transportation, cleaning, disinfection and preparation adding up to 40 min (30 min for Olympus turnover and 10 min for transport and assistance) for each intubation scope use in the operating room (151 intubations) and 70 min (30 min for Olympus turnover and 40 min for transport and assistance) for each intubation scope use in the remote location (15 intubations), the comparable turnover times in relation to billing personnel hours for the EndoSheath^®^ Technology Scopes are 5.44 min added to the 10-min for transport and assistance (15.44 min per equivalent 151 intubations done with Olympus scope in the operating room), and 5.44 min added to the 40-min for transport and assistance with the anesthesia technician personnel staying with the airway management team during the whole procedure (45.44 min per equivalent 15 intubations done with Olympus scope in the remote location). Hence the equivalent annual personnel costs for intubations with EndoSheath^®^ Technology Scopes are $703.20 and the personnel cost per intubation is $4.24 (703.2/166).

As compared to the mean 5.44 min turnover times of EndoSheath^®^ Technology Scopes, Olympus scope had been previously reported turnover times of 30-min for the cleaning and restocking the supplies. Therefore, the required number (adjusted to mean turnover time) of EndoSheath^®^ Technology Scopes to replace the six Olympus Scopes at our institute will be 1.09 [(5.44/30)(6)]. As the EndoSheath^®^ Technology Scope requirements should be a whole number, it will be most logical and tangible to replace the six Olympus Scopes at our institute with two EndoSheath^®^ Technology Scopes.

After the final procedural cost calculations based on two EndoSheath^®^ Technology Scopes, the previously reported data for Olympus[8] was reassessed to match up the current list prices: new Olympus reusable scope model LF-GP at $8,650.00 per scope and annual full maintenance contract for Olympus scope at $2,148.00 per scope (in our previous study[8], we had used the actual repairs data for the wear-tear over three years of our six Olympus scopes that cannot be compared with the EndoSheath^®^ Technology Scope as we do not have the actual repairs data for the EndoSheath^®^ Technology Scope) and the lifetime of Olympus scope was adjusted to 9-years to exclude the cost-bias against EndoSheath^®^ Technology Scope (9-years lifetime) as compared to the previously reported 15-year lifetime of Olympus scope per Olympus manufacture. Therefore, after reassessment, the intubation cost with six Olympus scopes increased from previously reported $119.75 to reassessed $158.50.

As described in ***Table 2***, even though the procedural costs with six EndoSheath^®^ Technology Scopes without adjustment for turnover times were significantly higher ($238.10; *P* = 0.007) in comparison to intubation costs of $158.50 with six Olympus Scopes, the costs of an airway related procedure using EndoSheath^®^ Technology Scope were significantly lower after adjustments for the turnover times [$81.50 with 1.09 EndoSheath^®^ Technology Scopes (*P* < 0.001), and $110.52 with two EndoSheath^®^ Technology Scopes (*P* = 0.009)].

## DISCUSSION

In our previously reported data analysis and interpretation[8], we had determined that fiberoptic intubations at our institute cost 119.75 USD (and after reassessment on the present study they cost 158.50 USD) with Olympus fiberscope, and had recommended that very high costs (approx. 300.00 USD) of single use fiberoptic intubation scope (Ambu Inc., Maryland, United States) may be deterrent to the replacement of the reusable Olympus Scope with single use intubation scope based on our institutional experience. However, the obvious advantages of the disposable scopes and their accessories in relation to prevention against the risks of cross-contamination and cross-infections associated with the reusable scopes prompted us (as researchers and as an institution) to re-explore the cost-effectiveness of the Olympus fiberoptic intubation scope that does not have any disposable accessories in contrast to the Vision Sciences, Inc., reusable fiberscope with single-use (disposable) EndoSheath^®^ Technology.

During our previously reported review of the Olympus fiberscope intubation costs at our institution, our major concerns were in relation to the prolonged and tedious disinfection processes involving the reusable scopes that act as deterrent to accelerate turnover times of the scopes between the cases in the operating room complexes or endoscopy suites or intensive care units that have rapid turnover of the clinical procedural cases. Additionally, despite the adequate disinfection, there is always a potential but differential risk of the transfer of the contaminants and pathogens entrapped in the suction channels of the insertion tube of the fiberscope whether the suction channels' integrity is intact or not. Moreover, the suction channels' breakage is the major contributor to the repair costs of the fiberscope because the hollow suction channels are also utilized as ports for the therapeutic endoscopic interventions/instrumentations. Vision Sciences, Inc., reusable fiberscope with single-use (disposable) EndoSheath^®^ Technology has innovated the technology of exteriorizing the suction channels by removing the suction channels from the insertion tube of the fiberscope and by incorporating the suction channels of varying sizes (to meet the varying needs of the endoscopic interventions) in the disposable single use sheaths (EndoSheath^®^ Technology). Secondary to these changes, it initially became apparent that the costs of using disposable EndoSheath^®^ Technology will be costlier as compared to reusable Olympus scope with no costly disposable parts; this is evident in the raw data elicited in the columns 4 and 5 of ***Table 2*** wherein the unadjusted cost per procedure with EndoSheath^®^ Technology was 238.10 USD as compared to 158.50 USD with Olympus scope. Herein, it is appreciated that the cost-effectiveness of the EndoSheath^®^ Technology can only be appreciated after adjusting for improved turnover time-elicited-equivalent numbers of Vision Sciences, Inc., reusable fiberscopes that will be required to adequately cover the institutional needs of airway procedures that were previously met with higher number of in-house Olympus fiberscopes.

Subsequently, the adjustments were initially based on the comparative mean turnover times for the two types of fiberscopes (5.44 min *versus* previously reported 30 min) that conveyed that our institution will require 1.09 Vision Sciences, Inc., reusable fiberscopes to replace the six Olympus fiberscopes to match our airway procedure related requirements. Based on these requirements, the cost-effectiveness of the Vision Sciences, Inc., reusable fiberscopes with EndoSheath^®^ Technology was elicited (***Table 2***), which was statistically significant (*P* < 0.001). Moreover, this statistical significance was adequately maintained (*P* = 0.009) when the equivalence number for Vision Sciences, Inc., reusable fiberscopes was logically converted into a whole number (= two) to calculate the more logical comparative procedural costs for EndoSheath^®^ Technology (110.52 USD). The logically converted number (= two) for EndoSheath^®^ Technology Scopes was also tangible for operating room personnel wherein the needs for airway related procedures at remote locations outside operating room suites would not leave a void for readily accessible fiberoptic apparatus in the operating room for airway procedures. Additionally, though the actual incidence rates of cross contamination and cross infection were not studied and analyzed in our study protocol, it will be prudent to state that lower costs per procedure with Vision Sciences, Inc., reusable fiberscopes may be worthwhile because their single-use accessories and the absence of the potential entrapping in-built suction channels in the insertion tubes of the reusable scopes can potentially prevent the unmeasured costs of cross-contamination and cross infection between the patients.

We have recognized some limitations of our study and EndoSheath^®^ Technology. The success rates of first attempt for airway procedures were low (83%), because all the procedures were performed by the Clinical year-1 and Clinical year-2 residents, and supervised by Clinical year-3 residents, and were abandoned in favor of alternate airway instruments for the subsequent attempts. It is important to realize that even though we use only three types of the single use (disposable sheaths) BF-0/BF-1.5/BF-2.1 EndoSheath^®^ Technology, the sheaths are also available in another (fourth) size (BF-2.8) and it is always helpful to decide on different sizes of the sheaths based on the requirement of the airway procedure. For instance, BF-0 sheaths without a suction channel were preferred for the smaller sized endotracheal tubes as well as for the endobronchial tube placement confirmations; BF-2.1 sheaths were preferred for bronchoscopy and broncho-alveolar lavage to provide wider lumen for the suction of thick and/or purulent tracheo-bronchial secretions. The good lubrication and proper alignment of the suction channel is essential to avoid the torsion on the sheath as it is advanced in the airway or is withdrawn from the airway to avoid the breaches in the sheath and the consequent need for traditional tedious and time consuming disinfection of the underlying contaminated fiberscope. It was our experience that as compared to the Olympus fiberscope with Snap-Up and Snap-Down latching mechanism to switch on/switch off the inbuilt miniature light source, the Vision Sciences, Inc., reusable fiberscope's miniature inbuilt miniature light source has to be screwed clockwise/anticlockwise for turning off/on the illumination and this twisting mechanism is sometimes tedious because of the plastic interface of EndoSheath^®^ between the operator's fingers and the underlying light source. The dry-cleaning of the Vision Sciences, Inc., reusable fiberscope before covering it with a new EndoSheath^®^ was essential so as to avoid the trapping of the moisture that will interfere with the adequate visualization of the airway structures as well as the appreciation of the breach in the EndoSheath^®^ after the completion of the procedure. In regards to the cost-effectiveness, the goals of much higher savings were interfered by the high purchasing costs of the Vision Sciences, Inc., reusable fiberscope (11,650.00 USD per scope) and high annual maintenance contract fees (4,000.00 USD annually per scope). Moreover, as the costs assessment were based on the low number (166) of annual airway related procedures performed by our anesthesia team, it needed to be seen the actual turnover times of the Vision Sciences, Inc., reusable fiberscope was 5.44 min as compared to 30 min with Olympus scope that would be more prudent and more cost-effective in much busier practice like bronchoscopy suites, intensive care units and other busier anesthesia care teams. The effect of multiple location airway services on the procedure costs may be resolved with the possibility of using the costly disposable scopes (approx. 300.00 USD) at the locations where the presence of these fiberscopes is only for the rare emergent airway scenario as compared to the elective and high volume fiberoptic intubations and related airway procedures in the operating rooms. There is always concern on how to test the breach in the used EndoSheath besides the visual appreciation of gross tear in the EndoSheath and the visual inspection for the moisture on the insertion cord after removing the used EndoSheath. The positive points that may go in favor of EndoSheath scope use are a) the absence of any suction channel in the insertion cord of the scope and hence the absence of the hidden hollow tube that harbors the contaminants and infectious agents in the reusable scopes; and b) the two lines of defense against cross contamination as removing the used EndoSheath and cleaning the EndoSheath scope, the EndoSheath scope is covered with a new EndoSheath under sterile conditions that provide the second line of defense against the cross-contamination. Moreover, though there are material, procedural and time standards for disinfection of the reusable scopes, the proficiency of the personnel who are executing these disinfecting procedures cannot be standardized until the universal implication of cost-prohibitive microbial flora assessment after each scope use (though how often the microbial flora identified on the insertion cord will actually cause cross-contamination cannot be yet quantified). However, if the care providers and operators want to be more thorough in appreciating the breaches in the used EndoSheath, they may consider testing the used EndoSheath with a colored solution like methylene blue injected into the inner side of the used EndoSheath after removing the sheath from the scope and then placing the used EndoSheath filled with colored solution in a fresh bowl or bottle of water to appreciate the change in the color of the water in cases of unidentified breaches.

In summary, based on our institutional experience, Vision Sciences, Inc., reusable fiberscope with EndoSheath^®^ Technology is significantly cost effective as compared to the Olympus Scope with significantly improved turnover times.
